# Decline in US–China science: Can ophthalmology remain collaborative?

**DOI:** 10.1016/j.aopr.2024.11.002

**Published:** 2024-11-06

**Authors:** Kevin Y. Huang, Parth A. Patel, Austin Huang, Allen C. Ho, Jost B. Jonas, Xiaodong Sun, Youxin Chen, Yingfeng Zheng, Yih-Chung Tham, Christina Y. Weng, Tien Yin Wong

**Affiliations:** School of Medicine, University of Texas Medical Branch, Galveston, TX, USA; School of Medicine, Medical College of Georgia, Augusta, GA, USA; School of Medicine, Baylor College of Medicine, Houston, TX, USA; Wills Eye Hospital, Philadelphia, PA, USA; Department of Ophthalmology, Medical Faculty Mannheim, Heidelberg University, Mannheim, Germany; Department of Ophthalmology, Shanghai General Hospital, School of Medicine, Shanghai Jiao Tong University, Shanghai, China; Department of Ophthalmology, Peking Union Medical College Hospital, Beijing, China; Zhongshan Ophthalmic Center, Sun Yat-sen University, Guangzhou, China; Singapore Eye Research Institute, Singapore National Eye Center, Singapore; Department of Ophthalmology, Yong Loo Lin School of Medicine, National University of Singapore, Singapore; Department of Ophthalmology, Cullen Eye Institute, Baylor College of Medicine, Houston, TX, USA; Singapore Eye Research Institute, Singapore National Eye Center, Singapore; Tsinghua Medicine, Tsinghua University, Beijing, China

Dear Editor,

While trends in scientific collaborations between the US and China have now been described in a general context^1^^,^^2^***—***revealing a significant decline in productivity compared to other international partnerships***—***such trends have yet to be specifically evaluated for the field of ophthalmology, where there is an ever-important emphasis on multinational partnerships for the delivery of state-of-the-art, rigorous, and equitable eye care.^1^^,^^3^, ^4^, ^5^ In this study, we explore collaborations between researchers in the US and China in ophthalmology-related literature from 2000 to 2021.

## 1 Methods

All original, peer-reviewed articles published between 2000 and 2021 listed under the category "Ophthalmology" were obtained from Thomson Reuters’ Web of Science (WoS) database. Alternative forms of publication, including meeting abstracts, letters, and book chapters, were excluded from the search. Articles published by authors affiliated with institutions located either in the US or China were extracted and stratified into three categories: those with US-based affiliations, those with affiliations in China, and those with affiliations in both the US and China. The number of citations for each of these articles was also assessed via the WoS database.

To assess productivity and impact in conjunction with crude publication and citation numbers, obtained via Thomson Reuters’ Web of Science (WoS) database, we calculated a modified Activity Index (AI), Attractivity Index (AAI), adjusted collaborative AI (AIc), and adjusted collaborative AAI (AAIc), defined as follows:

AI ​= ​{(N_ia_/N_ih_)/(N_ba_/N_bh_)}, where N_ia_ ​= ​number of publications from the US or from China in a given year; N_ih_ ​= ​total number of publications from both the US and China in a given year; N_ba_ ​= ​total number of publications from the US or China between 2000 and 2021; N_bh_ ​= ​total number of publications from both the US and China between 2000 and 2021.

AAI ​= ​{(S_ia_/S_ih_)/(S_ba_/S_bh_)}, where S_ia_ ​= ​number of citations on publications from the US or China in a given year; S_ih_ ​= ​total number of citations on publications from both the US and China in a given year; S_ba_ ​= ​total number of citations on publications from the US or China between 2000 and 2021; S_bh_ ​= ​total number of citations on publications from both the US and China between 2000 and 2021.

AIc ​= ​{(N_iac_/N_ihc_)/(N_bac_/N_bhc_)}, where N_iac_ ​= ​number of collaborative publications from the US and China in a given year; N_ihc_ ​= ​total number of publications from both the US and China in a given year; N_bac_ ​= ​total number of collaborative publications from the US and China between 2000 and 2021; N_bhc_ ​= ​total number of publications from both the US and China between 2000 and 2021.

AAIc ​= ​{(S_iac_/S_ihc_)/(S_bac_/S_bhc_)}, where S_iac_ ​= ​number of citations on collaborative publications from the US and China in a given year; S_ihc_ ​= ​total number of citations on publications from both the US and China in a given year; S_bac_ ​= ​total number of citations on collaborative publications from the US and China between 2000 and 2021; S_bhc_ ​= ​total number of citations on publications from both the US and China between 2000 and 2021.

Additionally, we compiled the top 100 most-cited articles with US-only, China-only, and US–China affiliations between 2000 and 2021 using the WoS database. To evaluate differences in the scope and the impact of research, we examined the data provided by the WoS database concerning the journal in which the articles were published, as well as the number of times each article has been cited. Furthermore, we characterized the subspecialty focus of the articles as one of the following: retina, glaucoma, cornea, neuro-ophthalmology, and oculoplastics.

## 2 Results

**Fig. 1:**
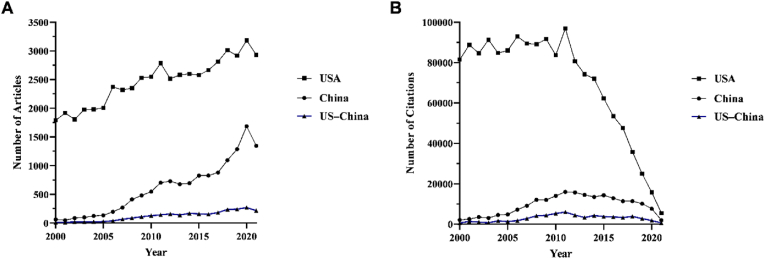
**Trends in output and citations between 2000 and 2021. (A)** Number of ophthalmology articles published between 2000 and 2021 by authors separated into three groups: China, US, and US–China. **(B)** Number of citations from ophthalmology articles published between 2000 and 2021 by authors separated into three groups: China, US, and US–China.

**Fig. 2:**
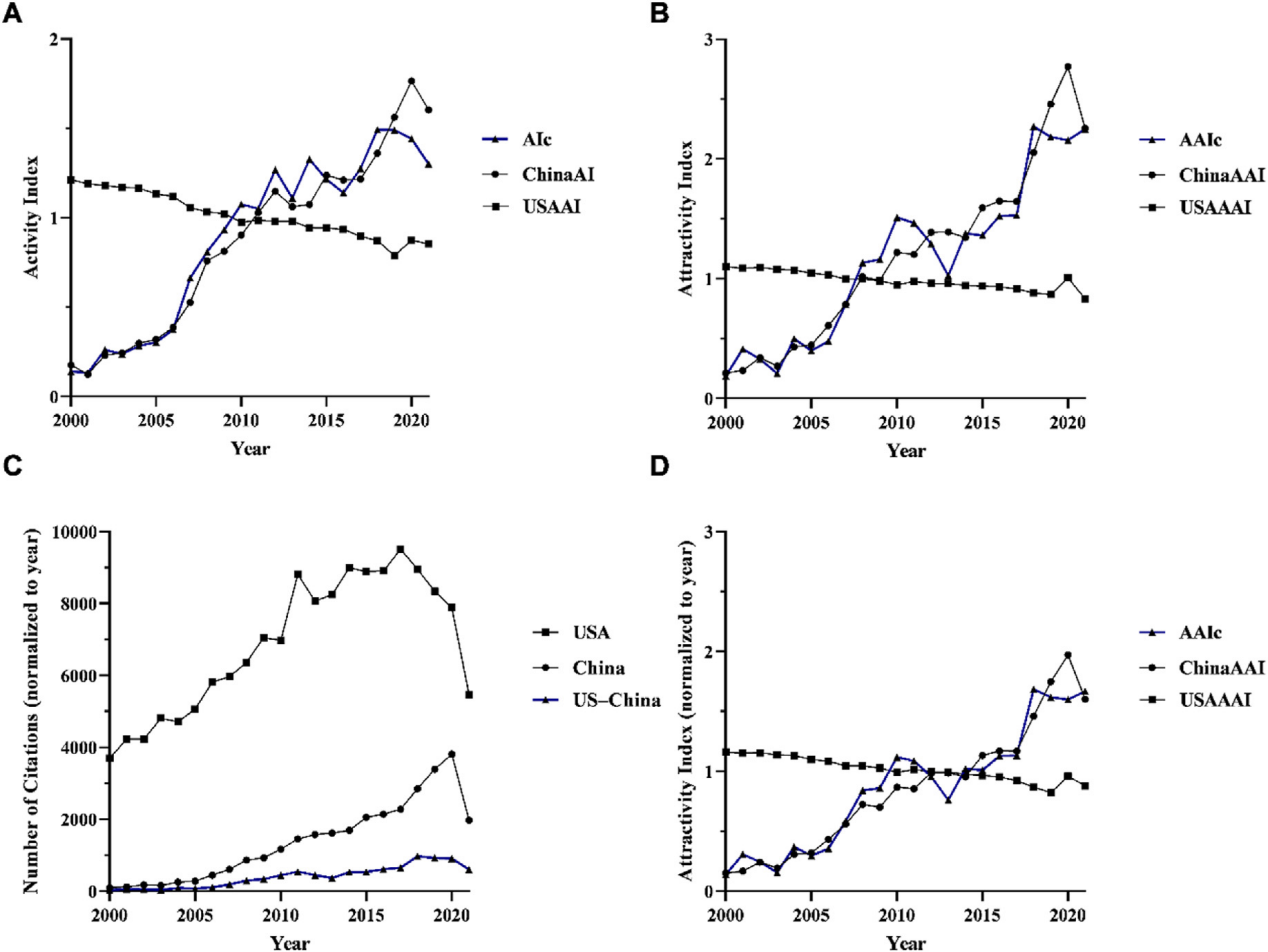
**Trends in Activity Index and Attractivity Index between 2000 and 2021. (A)** Activity Index (AI) of ophthalmology articles published between 2000 and 2021 by authors separated into three groups: China (ChinaAI), US (USAI), and US–China (AIc). AI ​= ​{(N_ia_/N_ih_)/(N_ba_/N_bh_)}, where N_ia_ ​= ​number of publications from the US or from China in a given year; N_ih_ ​= ​total number of publications from both the US and China in a given year; N_ba_ ​= ​total number of publications from the US or China between 2000 and 2021; N_bh_ ​= ​total number of publications from both the US and China between 2000 and 2021. **(B)** Attractivity Index (AAI) of ophthalmology articles published between 2000 and 2021 by authors separated into three groups: China (ChinaAAI), US (USAAI), and US–China (AAIc). AAI ​= ​{(S_ia_/S_ih_)/(S_ba_/S_bh_)}, where S_ia_ ​= ​number of citations on publications from the US or China in a given year; S_ih_ ​= ​total number of citations on publications from both the US and China in a given year; S_ba_ ​= ​total number of citations on publications from the US or China between 2000 and 2021; S_bh_ ​= ​total number of citations on publications from both the US and China between 2000 and 2021. **(C)** Number of citations from ophthalmology articles normalized by number of years since publication between 2000 and 2021 by authors separated into three groups: China, US, and US–China. **(D)** Attractivity Index (AAI) of ophthalmology articles normalized by number of years since publication between 2000 and 2021 by authors separated into three groups: China (ChinaAAI), US (USAAI), and US–China (AAIc).

Both the quantity of (Fig. 1A) and citations accrued by (Fig. 1B) ophthalmology-related literature from the US, China, and from US–China collaborations increased in the study period. Authors affiliated with the US amassed the greatest number of articles and citations. The decline in citations for all groups in the latter half of the study period can be attributed to the shorter time elapsed since the articles’ publication. The AI (Fig. 2A) and AAI (Fig. 2B) remained stagnant or slightly declined from 2000 to 2021 for research from the US, while these measures increased for research arising from US–China collaborations. These trends persisted even after normalizing the citation numbers (Fig. 2C) and the AAI (Fig. 2D) by the number of years since publication. The field of ophthalmology has, thus far, withstood the changes in cooperative dynamics that have impacted the broader scientific community.

Among the top 100 most-cited ophthalmology articles authored by researchers from the US and China, the journals *Investigative Ophthalmology & Visual Science*, *Ophthalmology*, and *JAMA Ophthalmology* were the most represented, with 35, 23, and 7 appearances, respectively. Sixteen percent of the top 100 most-cited ophthalmology articles from 2000 to 2021 arising from US–China collaborations had more than 250 citations, compared to 100% and 18% of the US-only and China-only affiliated research. Additionally, the articles were categorized by subspecialty focus (e.g., retina, glaucoma, cornea, neuro-ophthalmology, oculoplastics). Among this set of US–China collaborative articles, the retina subspecialty was the most represented, comprising 26% of the 100 most-cited articles from 2000 to 2021.

## 3 Discussion

Amid dwindling US–China collaborations in other scientific fields, partnerships in ophthalmology are on the rise. The AIc, a proxy for collaborative research output, and AAIc, a proxy for the relative research impact of collaborative publications, revealed a clear upward trajectory in US–China collaborations in ophthalmology research from 2000 to 2021. Even in the period from 2019 to 2021, marked by challenges arising from the COVID-19 pandemic, the AIc showed only a moderate decline, with the collaborative output score reverting to the value observed just two years prior, in 2017. The trend in AAIc is particularly noteworthy; the adjusted impact score showed no significant change from 2019 to 2021, while the broader scientific community saw a measurable decline in the impact-weighted productivity of US–China collaborations.^4^

The importance of collaborative efforts between the US and China in ophthalmology transcends scientific achievements alone. International partnerships have made possible the delivery of numerous ophthalmic grants to low- and middle-income countries, providing training and research opportunities in areas where the burden of eye disease is substantial, and access to ophthalmologists is limited.^3^ A remarkable success story in global eye care, the Afro-German-Eye-Net, Himalayan Cataract Project, and Orbis International continue to provide ophthalmology care and training across sub-Saharan Africa.^6^^,^^7^ In the Asia-Pacific region, the Global Eye Genetics Consortium, comprising researchers from the US, Hong Kong, Japan, and India, represents another such organization dedicated to researching rare genetic eye diseases affecting populations in Asia, Africa, South America, and the Middle East.^5^ Building on the successes of other international partnerships, ophthalmologists and scientists in the US and China have a shared vision of the future***—***one that embraces cooperative research efforts to push the boundaries of scientific achievement, for the good of health and humanity.

## Study approval

Not applicable.

## Author contributions

KYH, PAP, AH, CYW, and TYW designed and directed the research. PAP conducted descriptive statistical analysis on the dataset. KYH and PAP wrote the initial draft of the manuscript. KYH, PAP, AH ACH, YCT, JBJ, XS, YC, YZ, CYW, and TYW revised the manuscript. The authors collectively reviewed and approved the final manuscript.

## Funding

This work was undertaken without financial support, including but not limited to sponsorship from universities, charities, or commercial organizations.

## Declaration of competing interest

The authors declare that they have no known competing financial interests or personal relationships that could have appeared to influence the work reported in this paper.

## Data availability

The data presented in this study are available at https://figshare.com/s/9a1878980e56c96f553b.
